# Assessment of Ki‐67 and mitoses in pituitary neuroendocrine tumours—Consistency counts

**DOI:** 10.1111/bpa.13285

**Published:** 2024-07-15

**Authors:** Paul Benjamin Loughrey, Christine Greene, Kris D. McCombe, Fatima Abdullahi Sidi, Stephen McQuaid, Stephen Cooke, Steven J. Hunter, Brian Herron, Márta Korbonits, Stephanie G. Craig, Jacqueline A. James

**Affiliations:** ^1^ Patrick G Johnston Centre for Cancer Research Queen's University Belfast Belfast UK; ^2^ Regional Centre for Endocrinology and Diabetes Belfast Health and Social Care Trust Belfast UK; ^3^ Northern Ireland Biobank Queen's University Belfast Belfast UK; ^4^ Department of Neurosurgery Belfast Health and Social Care Trust Belfast UK; ^5^ Department of Cellular Pathology Belfast Health and Social Care Trust Belfast UK; ^6^ Department of Endocrinology William Harvey Research Institute, Barts and the London School of Medicine, Queen Mary University of London London UK

**Keywords:** digital pathology, Ki‐67, mitotic index, neuropathology, pituitary adenoma, pituitary neuroendocrine tumour

## Abstract

Pituitary neuroendocrine tumour Ki‐67 proliferation index varies according to the number of tumour cells assessed. Consistent Ki‐67 scoring approaches and re‐evaluation of the recommended Ki‐67 3% cut‐off are required to clarify controversies in pituitary neuroendocrine tumour Ki‐67 proliferation index assessment.

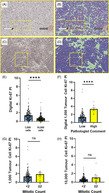

Symptomatic pituitary neuroendocrine tumours (PitNETs) are common, occurring in approximately 1 in 1100 of the general population and they account for 16.4% of central nervous system and intracranial tumours [1, 2]. Despite the prevalence of PitNETs, there is a lack of reliable biomarkers with utility in predicting aggressive features. Ki‐67 proliferative index (PI) and mitotic counts have traditionally been used in attempts to prognosticate PitNETs; however, the most recent World Health Organization (WHO) classification has attributed little value to these biomarkers [3]. Updated European Society of Endocrinology guidelines for aggressive pituitary tumours are currently under revision.

Despite guidelines for the assessment of the Ki‐67 PI and mitotic figures, the methodology employed to assess them has varied widely [4, 5]. This raises questions about the consequent cut‐offs currently employed in clinical practice.

The aims of this study were to investigate how Ki‐67 PI varies with the number of PitNET cells assessed, how manual semi‐quantitative Ki‐67 scoring recorded in a neuropathology report compares with scoring using digital image analysis (DIA) and how digital Ki‐67 PI and digital mitotic index may correlate. The secondary aims were to assess how Ki‐67 PI and mitotic counts, when determined using DIA, correlate with tumour size, tumour invasiveness, anterior pituitary hormone expression and inferred transcription factor status.

Whole‐face sections of PitNETs created as part of standard care were retrieved from tissue archives. The tissue had been stained with the Dako Ki‐67 MIB‐1 clone (catalogue number M7240). These slides were scanned at ×40 magnification (0.25 μm/pixel) using *Aperio AT2* (Leica Biosystems, Vista, CA, USA) and analysed with QuPath DIA software version 0.2.3 (Figure 1A–D) [6]. For mitotic index assessment, new sections of formalin‐fixed paraffin‐embedded PitNET samples were cut at 3 μm thickness and stained with haematoxylin alone to make mitotic figures more readily identifiable. A script was run to generate 10 circles of 400 μm diameter to replicate areas of 10 high‐powered fields at ×400 microscopic field (×40 objective) within QuPath. Mitotic figures were manually counted within these regions. All clinical parameters were retrieved from a retrospectively generated clinicopathological database. Statistical analysis was undertaken using *GraphPad Prism* software version 10.2.0.

**FIGURE 1:**
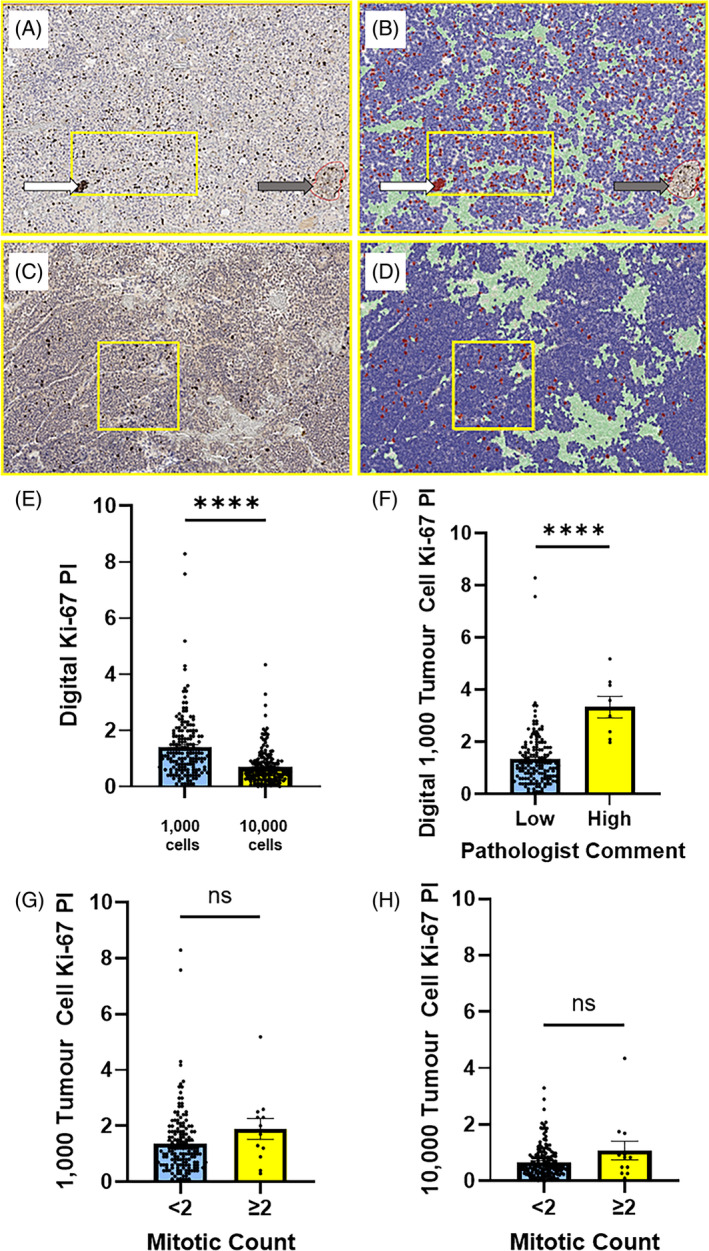
(A) MIB‐1‐stained PitNET without QuPath mask. Large yellow box indicates 10,000 PitNET cells (Ki‐67 PI 9.9%), small box indicates 1000 PitNET cells (Ki‐67 PI 17.7%). In this case, a clear hotspot of Ki‐67 expression is seen (white arrow). Artefact omitted from analysis shown using grey arrow. Image ×7 magnification. (B) The same MIB‐1‐stained PitNET indicated in (A). with QuPath mask. Large yellow box indicates 10,000 PitNET cells (Ki‐67 PI 9.9%), small box indicates 1000 PitNET cells (Ki‐67 PI 17.7%). In this case, a clear hotspot of Ki‐67 expression is seen (white arrow). Artefact omitted from analysis shown using grey arrow. Ki‐67 positive tumour cells red, Ki‐67 negative tumour cells blue, stromal cells and blood cells green. Image ×7 magnification. (C) MIB‐1‐stained PitNET without QuPath mask. Large yellow box indicates 10,000 PitNET cells (Ki‐67 PI 1.8%), small box indicates 1000 PitNET cells (Ki‐67 PI 3.6%). In this case, distinct areas of Ki‐67 hotspot expression are less evident. Image ×7 magnification. (D) The same MIB‐1‐stained PitNET indicated in (C) with QuPath mask. Large yellow box indicates 10,000 PitNET cells (Ki‐67 PI 1.8%), small box indicates 1000 PitNET cells (Ki‐67 PI 3.6%). Ki‐67 positive tumour cells red, Ki‐67 negative tumour cells blue, stromal cells and blood cells green. Image ×7 magnification. (E) Box plot comparing Ki‐67 PI obtained when assessing 1000 PitNET cells (median 1.19%) compared to assessing 10,000 PitNET cells (median 0.50%), *n* = 174, Wilcoxon matched‐pairs signed rank test, ****p < 0.0001. (F) Box plot comparing digital Ki‐67 in PitNETs assessed as having low Ki‐67 PI (median 1.10%) or high (median 3.30%) according to neuropathologist comment. Neuropathologist assessment was semi‐quantitative measured against traditional cut‐off of ≥3%, that is, low: <3%, high: ≥3%, *n* = 166, data unavailable for eight cases, Mann–Whitney test, ****p < 0.0001. (G) Box plot showing no significant difference in Ki‐67 PI when assessing Ki‐67 1000 cell PI in tumours with mitotic counts <2 (median 1.10%) and ≥2 (median 1.85%), *n* = 174, Mann–Whitney test, ns: not significant, *p* = 0.090. (H) Box plot showing no significant difference in Ki‐67 PI when assessing Ki‐67 10,000 cell PI in tumours with mitotic counts <2 (median 0.50%) and ≥2 (median 0.83%), *n* = 174, Mann–Whitney test, ns: not significant, *p* = 0.199.

One hundred seventy‐four MIB‐1‐stained whole‐face sections from primary surgeries were assessed for digital Ki‐67 and mitotic figures. The Ki‐67 PI when assessing 1000 PitNET cells was significantly higher than when assessing 10,000 PitNET cells (*p* < 0.001, Figure 1E). When comparing digital 1000 PitNET cell Ki‐67 PI with a reporting consultant neuropathologist, there was a significant difference when semi‐quantitative assessment was dichotomised as high or low (*p* < 0.001, Figure 1F). There was no significant difference between digital Ki‐67 PI for 1000 tumour cell assessment or 10,000 cell assessment stratified by mitotic count cut‐off of 2 (*p* = 0.090, Figure 1G and *p* = 0.199, Figure 1H).

Digital Ki‐67 PI for 1000 tumour cells and digital mitotic counts were not significantly different between micro‐PitNETs and macro‐PitNETs (*p* = 0.059, Figure 2A and *p* = 0.752, Figure 2B). When considering tumour invasiveness, digital Ki‐67 PI for 1000 tumour cells was significantly higher in non‐invasive tumours (tumours without radiological evidence of sphenoid and/or cavernous sinus invasion) (*p* = 0.044, Figure 2C). Digital mitotic index was not significantly different between invasive and non‐invasive PitNETs (*p* = 0.941, Figure 2D). There were significant differences in digital Ki‐67 PI when tumours were assessed according to anterior pituitary hormone expression (*p* < 0.001, Figure 2E). Transcription factor status was unknown because of the retrospective nature of the cohort; however, when inferring transcription factor status (Figure 2F), there was no significant difference in the digital Ki‐67 PI between PIT1, SF‐1 and TPIT subgroups (*p* = 0.681, Figure 2G).

**FIGURE 2:**
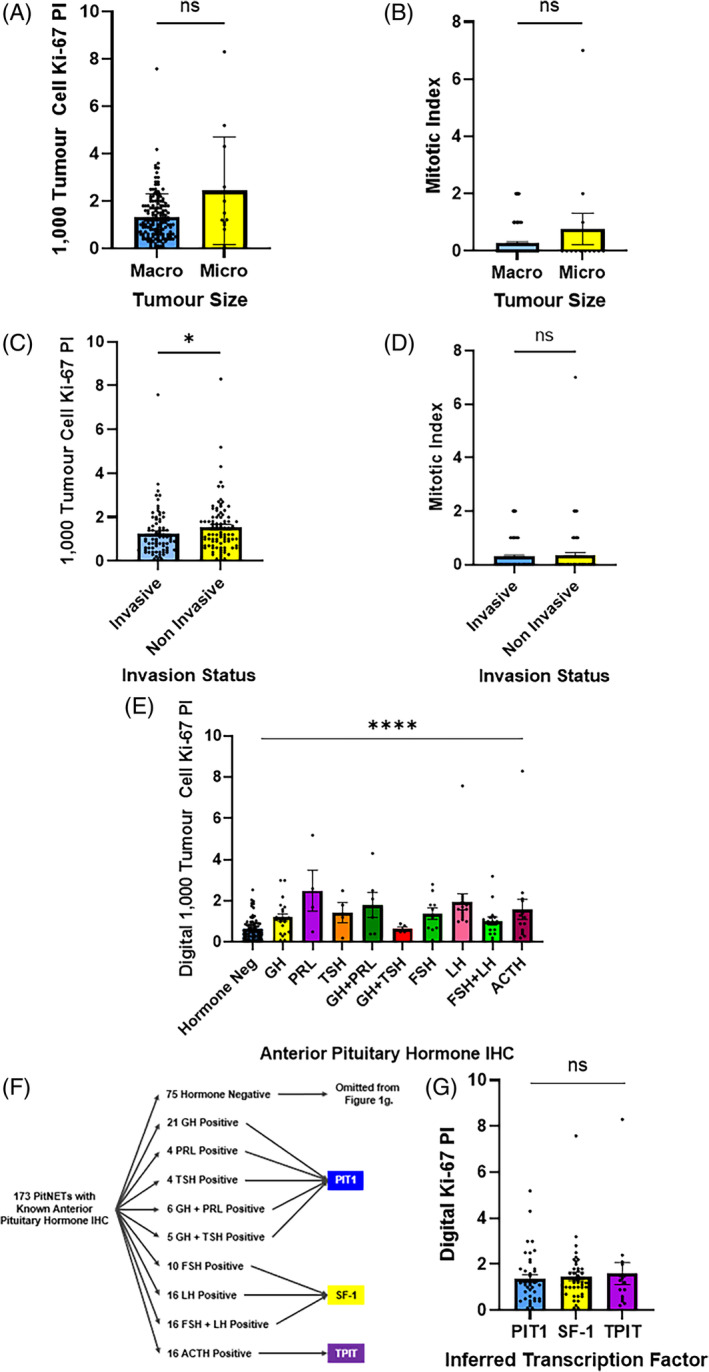
(A) Box plot showing no significant difference in digital Ki‐67 PI in macro‐PitNETs (median 1.10%) compared to micro‐PitNETs (median 1.50%), *n* = 173, one unknown, Mann–Whitney test, ns: not significant, *p* = 0.059. (B) Box plot showing no significant difference in digital mitotic count in macro‐PitNETs (median 0) compared to micro‐PitNETs (median 0), *n* = 173, one unknown, Mann–Whitney test, ns: not significant, *p* = 0.752. (C) Box plot showing non‐invasive PitNETs had significantly higher Ki‐67 PI (median 1.2%) compared to invasive PitNETs (median 1.0%), *n* = 169, five unknown, Mann–Whitney test, *p* = 0.044. (D) Box plot showing no significant difference in digital mitotic count in invasive (median 0) compared to non‐invasive (median 0) PitNETs, *n* = 169, five unknown, Mann–Whitney test, *p* = 0.941. (E) Box plot of Ki‐67 PI according to anterior pituitary hormone immunohistochemical (IHC) analysis expression of the cohort, *n* = 173, one tumour data unavailable, Kruskal–Wallis test, ****p < 0.0001. (F) Method employed to infer transcription factor status. Seventy‐five tumours were hormone‐negative and so were omitted from analysis in (G). The remainder of tumours were subclassified as PIT1, SF‐1 and TPIT. (G) Box plot of digital Ki‐67 PI according to inferred transcription factor lineage, *n* = 98, Kruskal–Wallis test, *p* = 0.681.

The assessment of Ki‐67 PI and mitotic counts have become routine in PitNET histological assessment, and a Ki‐67 PI cut‐off of ≥3% is cited [4, 5]. Despite this, limitations in the clinical utility of PitNET proliferative assessment are recognised, most notably in the 2022 WHO classification [3]. Identification of mitotic figures is difficult and open to subjectivity. There is also variation in the pituitary literature with regard to the techniques employed in Ki‐67 PI and mitotic figure assessment.

The results of this study demonstrate that the values of Ki‐67 PI obtained vary dependent on the number of PitNET cells that are assessed. These results align with recent expert appraisal which advocates for more accurate quantification of Ki‐67 PI [3]. Whilst this study was not designed to determine optimal prognostic Ki‐67 cut‐offs, previous work which contributed much of the evidence towards the traditional Ki‐67 cut‐off of ≥3% for PitNETs assessed an average of 4200 PitNET cells in 77 PitNETs [7]. Given that the results of the current study indicate an inverse relationship between PitNET cells assessed and Ki‐67 PI obtained, a cut‐off of ≥3% in an average of 4200 cells may translate to a higher Ki‐67 cut‐off for the assessment of 500–1000 tumour cells traditionally recommended in older guidelines and currently still employed in clinical practice. In the current study, Ki‐67 was significantly higher in non‐invasive tumours (Figure 2C). However, invasion was defined by preoperative imaging and therefore Ki‐67 PI appears to add little to that which would already be known in a patient who has undergone pituitary surgery.

The strengths of the work presented here are the relatively large number of samples representative of 8 consecutive years of unselected surgeries from a regional neurosurgical centre. The tissues analysed were taken from a heterogeneous population of PitNETs. The whole‐face sections utilised were stained using a MIB‐1 antibody clone in accordance with guidelines and were not optimised prior to DIA. Thus, they are representative of whole‐face sections neuropathologists work with in clinical practice. All digital assessments were undertaken by a single operator and underwent quality control with a consultant histopathologist. Furthermore, QuPath is an open‐access DIA software which has been used in over 200 publications to support the assessment of Ki‐67 PI. Consequently, the study should be reproducible.

Limitations of the current work include the retrospective nature which could contribute to variable staining intensity associated with age of samples at retrieval. Due to this retrospective nature, subgroup analysis according to transcription factor immunohistochemistry was not available. The results in this study show mitotic figures to be infrequent in PitNETs; however, assessment of digital images can result in underestimation of mitotic counts [8]. Finally, while QuPath can perform well, provide insights into DIA assessments and is comparable to clinically approved software, it is a research tool and is not clinically validated.

In conclusion, the literature indicates heterogeneous approaches to proliferative assessments in PitNETs. Although the 2022 WHO classification attributes little value to proliferative markers in PitNETs, in practice, many pituitary services continue to use them to inform clinical decision‐making. Data from this study suggest there is an inverse relationship between the number of PitNET cells assessed and the Ki‐67 PI obtained. Digital Ki‐67 PI and digital mitotic counts may have limited clinical value, even when using accurate quantification strategies. Therefore, consistent with the 2022 WHO classification, their efficacy as a stand‐alone tool for histopathological assessment and prognostication of PitNETs is doubtful. This has implications on clinical decision‐making for those multidisciplinary teams managing patients with PitNETs and continuing to rely on proliferative assessments. Consistent Ki67 scoring approaches and re‐evaluation of the recommended Ki‐67 PI 3% cut‐off are required to clarify this controversial topic.

## CONFLICT OF INTEREST STATEMENT

The authors declare no conflicts of interest.

## ETHICS STATEMENT

The human samples in this study were provided by the Northern Ireland Biobank (NIB). NIB is a Human Tissue Authority (HTA) Licenced Research Tissue Bank with generic ethical approval from The Office of Research Ethics Committees Northern Ireland (ORECNI REF 21/NI/0019). Ethical approval for access to and use of these samples in research was granted by the NIB (application reference NIB18‐0282). Informed consent was not required for this retrospective study of anonymised samples and data.

## Data Availability

The data sets used and/or analysed during the current study are available from the corresponding author on reasonable request.
